# Walking With Horizontal Head Turns Is Impaired in Persons With Early-Stage Multiple Sclerosis Showing Normal Locomotion

**DOI:** 10.3389/fneur.2021.821640

**Published:** 2022-01-28

**Authors:** Ilaria Carpinella, Elisa Gervasoni, Denise Anastasi, Rachele Di Giovanni, Andrea Tacchino, Giampaolo Brichetto, Paolo Confalonieri, Claudio Solaro, Marco Rovaris, Maurizio Ferrarin, Davide Cattaneo

**Affiliations:** ^1^IRCSS Fondazione Don Carlo Gnocchi, Milan, Italy; ^2^Centro di Recupero e Rieducazione Funzionale (CRRF) Mons. Luigi Novarese, Moncrivello, Italy; ^3^Italian Multiple Sclerosis Foundation, Scientific Research Area, Genoa, Italy; ^4^IRCCS Foundation “Carlo Besta” Neurological Institute, Milan, Italy; ^5^Department of Physiopathology and Transplants, University of Milan, Milan, Italy

**Keywords:** multiple sclerosis, wearable inertial sensors, instrumented assessment, dynamic balance, rehabilitation outcome assessment

## Abstract

**Background:**

Turning the head while walking (an action often required during daily living) is particularly challenging to maintain balance. It can therefore potentially reveal subtle impairments in early-stage people with multiple sclerosis who still show normal locomotion (NW-PwMS). This would help in identifying those subjects who can benefit from early preventive exercise aimed at slowing the MS-related functional decline.

**Objectives:**

To analyze if the assessment of walking with horizontal head turns (WHHT) through inertial sensors can discriminate between healthy subjects (HS) and NW-PwMS and between NW-PwMS subgroups. To assess if the discriminant ability of the instrumented WHHT is higher compared to clinical scores. To assess the concurrent validity of the sensor-based metrics.

**Methods:**

In this multicenter study, 40 HS and 59 NW-PwMS [Expanded Disability Status Scale (EDSS) ≤ 2.5, disease duration ≤ 5 years] were tested. Participants executed Item-6 of the Fullerton Advanced Balance scale-short (FAB-s) wearing three inertial sensors on the trunk and ankles. The item required to horizontally turn the head at a beat of the metronome (100 bpm) while walking. Signals of the sensors were processed to compute spatiotemporal, regularity, symmetry, dynamic stability, and trunk sway metrics descriptive of WHHT.

**Results:**

Mediolateral regularity, anteroposterior symmetry, and mediolateral stability were reduced in NW-PwMS vs. HS (*p* ≤ 0.001), and showed moderate discriminant ability (area under the receiver operator characteristic curve [AUC]: 0.71–0.73). AP symmetry and ML stability were reduced (*p* ≤ 0.026) in EDSS: 2–2.5 vs. EDSS: 0–1.5 subgroup (AUC: 0.69–0.70). The number of NW-PwMS showing at least one abnormal instrumented metric (68%) was larger (*p* ≤ 0.002) than the number of participants showing abnormal FAB-s-Item6 (32%) and FAB-s clinical scores (39%). EDSS: 2–2.5 subgroup included more individuals showing abnormal instrumented metrics (86%) compared to EDSS: 0–1.5 subgroup (57%). The instrumented metrics significantly correlated with FAB-s-Item6 and FAB-s scores (|Spearman's *r*_*s*_| ≥ 0.37, *p* < 0.001), thus demonstrating their concurrent validity.

**Conclusion:**

The instrumented assessment of WHHT provided valid objective metrics that discriminated, with higher sensitivity than clinical scores, between HS and NW-PwMS and between EDSS subgroups. The method is a promising tool to complement clinical evaluation, and reveal subclinical impairments in persons who can benefit from early preventive rehabilitative interventions.

## Introduction

The head is a natural reference frame for movement since it contains the visual and vestibular systems indispensable to correctly detect self-motion in space (1). Since head stabilization during movement is of paramount importance to optimize the functioning of these sensory systems, head oscillations during natural walking are kept minimal (around 2°) (2). On the other hand, common daily-life actions, such as crossing a street or talking with a friend during a stroll, require walking with larger horizontal head rotations.

Moving the head during locomotion naturally challenges the balance control system since it requires the accurate integration of vestibular, visual, and proprioceptive information to modulate the vestibulo-ocular and vestibulospinal reflexes responsible for gaze stabilization/redirection and dynamic balance maintenance (3, 4). Consequently, walking with horizontal head turns (WHHT) is particularly difficult for individuals showing vestibular dysfunction (4, 5), and/or deficits in sensory processing and integration commonly present in people with multiple sclerosis (PwMS) (6, 7). Previous studies on PwMS with moderate-to-severe mobility impairment showed that WHHT was abnormal in 80% of participants (8) and represented the most difficult item of the Dynamic Gait Index (DGI) (9). Importantly, WHHT, as measured by the Fullerton Advanced Balance scale-short version (10), resulted to be the most impaired item (together with turning 360°) also in early-stage PwMS (11). Recently, Cattaneo et al. (12) found that WHHT is more impaired in PwMS compared to stroke survivors and people with Parkinson's disease, complementing previous results showing more severe static and dynamic balance deficits in PwMS (7, 13, 14).

Considering its high impact on dynamic balance maintenance, turning the head while standing or walking is included in several rehabilitation programs (6, 15–17) and clinical assessment scales, such as the DGI (9), the MiniBESTest (18), and the Fullerton Advanced Balance scale (19) and its short version (10). Although widely used, these evaluation tools may suffer from ceiling effect, limited sensitivity, and poor details in assessing different aspects of a task (20). These limitations may be partly overcome by wearable inertial measurement units (IMUs) which allow easy objective assessments of a motor task outside dedicated labs (20). Previous studies on PwMS have shown that IMU-based assessments may provide additional information about *how* a task is performed (21) through indexes more responsive to subtle impairments (22), disease progression (20, 23), and rehabilitation effects (24).

While most literature refers to natural walking and Timed Up and Go (TUG) test (20), no studies exist about the instrumented assessment of WHHT in early-stage non-disabled PwMS. Given the complexity of this task in terms of load on the sensorimotor system and considering that the sensory symptoms represent the first clinical manifestation of MS in 43% of patients (25), it can be hypothesized that the instrumented assessment of WHHT could detect subclinical motor impairments even in the early stages of MS when natural walking (i.e., walking with no imposed head rotations) is still normal. This would be of paramount importance to follow the course of these impairments and to identify, from the very early stages of the disease, those individuals who could benefit from preventive rehabilitation exercises, potentially useful to slow the MS-related functional decline, as recently indicated (26).

This multicenter cross-sectional study aims at analyzing the discriminant ability and the concurrent validity of an IMU-based assessment of WHHT in early-stage PwMS with normal natural walking (NW-PwMS). We hypothesized that the instrumented assessment of WHHT (i) can discriminate between healthy subjects and NW-PwMS, and between NW-PwMS subgroups, (ii) its discriminant ability is higher compared to clinical scales, and (iii) provides valid indexes to complement clinical assessments of WHHT and dynamic balance in early-stage PwMS.

## Methods

### Participants

A total of 82 consecutive PwMS [age, mean ± SD (range): 39.5 ± 10.6 (20–64) years; % females: 65.9%] were enrolled from three clinical Italian centers in Milan, Turin, and Genoa. Inclusion criteria were: age ≥ 18 years, MS diagnosis based on McDonald criteria (27), disease duration ≤ 5 years, and Expanded Disability Status Scale (EDSS) (28) ≤ 2.5. Exclusion criteria were: increase ≥ 1 in EDSS score over the last 3 months, diagnosis of major depression, severe joint and/or bone disorders interfering with balance and gait (based upon clinical judgment), and cardiovascular or other concomitant neurological diseases.

A total of 40 healthy subjects (HS) without any musculoskeletal or neurological disorders (age: 39.0 ± 10.9 years, 28 females) were also recruited. All the participants signed a written informed consent to the study that was approved by the local ethical committee of each center (approval numbers, Milan: 21/2017/CE_FdG/FC/SA; Turin: AslVC.CRRF.17.03; Genoa: 026/2018).

### Selection of Normal-Walking PwMS

People with multiple sclerosis were assessed with the Timed 25-foot Walk test (T25FWT) and with an IMU-based instrumented gait test.

The T25FWT measures the time taken to walk at maximum speed along a 7.62-m linear course (29). Participants presenting T25FWT scores above the normative cut-off [5.2 s (29)] were excluded from the subsequent analyses. The cut-off value of 5.2 s was chosen as it was the maximum T25FWT score [median (range): 3.7 (2.8–5.2) s] found by Phan-Ba et al. (29) in a sample of 104 healthy subjects with an age range (18–60 years) and sex distribution (% females: 63.5%) similar to those of the PwMS here recruited.

The remaining participants were required to walk a 15-m straight corridor at their maximum speed wearing three IMUs (MTw, Xsens, The Netherlands) above lateral malleoli and on the lower back. The latter position was chosen as it is the most widely used during gait tests, as described in the review by Vienne-Jumeau et al. (20). Signals of IMUs related to the middle five strides were processed (30) to compute three parameters commonly impaired in early-stage PwMS: cadence, stance time, and double-support time (23, 31). Since the present sample of forty HS did not execute the above test, the data of each patient were compared to the normative ranges collected from another group of 21 healthy volunteers (NORM) recruited in our previous studies. The NORM sample had age and sex distribution (age: 36.4 ± 8.8 years, % females: 66.7%) comparable to those of the PwMS here analyzed, and performed the straight-line walking test wearing the same sensors of the PwMS and following the same protocol. In particular, both groups were required to walk for 15 m at their maximum speed. PwMS showing at least one instrumented parameter outside the normative ranges were excluded, while the other ones were labeled as normal-walking PwMS (NW-PwMS) and underwent subsequent analyses.

### Clinical Assessment

In addition to the T25FWT and the 15-m instrumented test, the following clinical assessments were administered to NW-PwMS: the 12-item Multiple Sclerosis Walking Scale (MSWS-12) and the Fullerton Advanced Balance-short Scale (FAB-s). FAB-s was administered also to HS.

The MSWS-12 is a patient-reported questionnaire on walking ability. The questions focused on the self-perceived impact of MS on 12 daily-life locomotor activities in the last 2 weeks. The transformed total score is between 0 and 100, with higher scores indicating higher perceived walking difficulties (32, 33). The FAB-s measures dynamic balance during 6 tasks of daily living. Each item is rated on a 5-point (0–4) ordinal scale, with higher scores indicating better performances. Scores <23 are considered abnormal (10).

### Instrumented Assessment—WHHT

Healthy subjects (HS) and NW-PwMS were equipped with three wireless IMUs (MTw, Xsens, The Netherlands) secured on both shanks (above lateral malleoli) and the sternum. The position of the latter IMU was chosen to better describe sway and possible instability of the upper trunk that, based on our clinical experience, seem to occur more frequently during locomotor tasks particularly demanding in terms of dynamic balance [e.g., TUG test (34), walking while turning the head (13), walking around/over obstacles (13), stairway walking (14)], than during straight-line walking. IMU-derived accelerations and angular velocities were recorded at 75 Hz. Participants performed Item 6 of FAB-s (i.e., walk with horizontal head turns) following published instructions (19). In particular, a metronome was set to 100 bpm. Participants practiced horizontal head turns of 30° at the rhythm of the metronome while standing in place. When they felt ready, they walked along a 9-m straight path while turning their head from side to side at the metronome beat.

Trunk anteroposterior (AP), mediolateral (ML), and vertical (VT) accelerations were reoriented to a horizontal-vertical coordinate system (35). Heel-strike and foot-off instants were identified (30), and data related to the middle five strides [10 steps as indicated by the FAB instructions (19)] were used to compute 12 metrics organized in gait domains as described in Table 1.

**Table 1:** Description of the instrumented metrics.

**Domain**	**Metric**	**Description**
Spatiotemporal	Gait Speed (m/s)	The ratio between the pathway's length and the time taken to walk it.
	Cadence (stride/min)	Computed as 60/*T*_stride_, where *T*_stride_ is the stride duration (i.e., the time interval between two consecutive heel-strikes of the same foot).
	Stance duration (%)	Time interval between the instants of heel-strike and toe-off of the same foot, expressed as a percentage of *T*_stride_
	Double-Support Duration (%)	Time interval between the instants of heel-strike of one foot and the toe-off of the contralateral foot, expressed as a percentage of *T*_stride_
Regularity	AP and ML Stride Regularity (-)	The second peak of the normalized autocorrelation function computed from the trunk AP and ML acceleration components (40). Increasing values, from 0 to 1, indicate higher stride regularity.
Symmetry	AP and ML improved Harmonic Ratio (iHR) (%)	The trunk AP and ML acceleration signals were decomposed into harmonics using a discrete Fourier transform. Hence, iHR was computed as the percentage ratio between the sum of the powers of the first 10 in-phase harmonics to the sum of the powers of the first 20 (in-phase and out-of-phase) harmonics (41). Increasing values, from 0 to 100%, indicate more symmetrical gait.
Dynamic Stability	AP and ML short-term Lyapunov exponent (sLyE) (-)	sLyE reflects the ability of the locomotor system to manage small perturbations naturally occurring during walking, such as external mechanical disturbances or internal control errors (42). Trunk AP and ML acceleration signals related to five consecutive strides in the central part of the pathway were re-sampled to 5 × 100 frames to maintain equal data length across subjects. sLyE is estimated from each acceleration segment following Rosenstein method (43). In summary an *m-*dimensional state-space (*m* = 5) was reconstructed from each acceleration component and its delayed copies (delay *T* = 10 samples). The values of *m* and *T* parameters were estimated using published algorithms (44). The mean divergence curve (D) of the acceleration trajectories in the state-space was computed, and sLyE was calculated as the slope of the log(*D*) between 0 and 0.5 stride (1 step). Increasing values of sLyE (i.e., faster trajectory divergence) indicate a lower ability of the motor system to cope with small perturbations, thus reflecting lower dynamic stability.
Trunk Sway	AP and ML Normalized Trunk Acceleration (-)	SD of trunk AP and ML acceleration normalized with respect to the SD of the acceleration modulus. Increased values of this parameter indicate larger trunk sway, independently from gait speed (45).

*AP, anteroposterior; ML, mediolateral*.

The same parameters were computed also from the instrumented gait test executed during the screening procedure, although the position of the trunk sensor was different (low back). This was done (i) to make sure that the NW-PwMS actually walked normally, not only in terms of spatiotemporal aspects, and (ii) to allow comparisons with previous literature that have analyzed straight-line gait of early-stage PwMS using a sensor on the low back (36–39). Data processing was performed using MATLAB R2017b (The MathWorks, MA, USA).

### Statistics

Non-parametric statistics were used since data were not normally distributed (Shapiro–Wilk's test < 0.05). HS and NW-PwMS were compared using the chi-squared test (χ^2^) for sex, and the Mann–Whitney *U*-test for all the other clinical and instrumented features. Bonferroni–Holm (BH) correction for multiple comparisons was applied. The discriminant ability of each parameter was assessed by computing the area under the receiver operating characteristic curve (AUC). Only those parameters showing a statistically significant difference between HS and NW-PwMS were further analyzed. This subset of metrics was compared among HS, NW-PwMS with EDSS: 0–1.5, and NW-PwMS with EDSS: 2–2.5 using Kruskal–Wallis (KW), and Bonferroni–Holm *post-hoc* tests. The number of NW-PwMS showing abnormal values of the selected instrumented metrics was compared with the number of participants showing abnormal clinical scores using the chi-squared test. A parameter was considered abnormal if it was above the 95th (or below the 5th) percentile of HS values, depending on if its increase (or decrease) was indicative of poorer performances.

Concurrent validity of the instrumented metrics was assessed through Spearman's correlation coefficient (*r*_*s*_) with FAB-s, FAB-s-Item6, and MSWS-12 scores. The same method was used to evaluate the correlation among instrumented features. Statistical analyses were performed using STATISTICA (Statsoft, OK, USA).

## Results

### Sample Description

From the recruited sample of PwMS (*n* = 82), 22 were excluded because they showed T25FWT scores above the normative cut-off and/or because they presented at least one temporal aspect of instrumented natural walking outside the normative range. One participant was excluded since his/her instrumented data were corrupted. The remaining 59 participants (72%) were considered as normal-walking PwMS (NW-PwMS). The sample size (40 HS and 59 NW-PwMS) was considered adequate based on previous results on healthy subjects and early-stage PwMS (11) showing a mean between-group difference in the FAB-s Item 6 score of 0.6 ± 0.9 points (effect size: 0.66). These data indicated that 39 subjects per group were necessary to obtain a difference between groups with α = 0.05 and Power (1-β) = 0.80.

As shown in Table 2, NW-PwMS included 37 participants with EDSS: 0–1.5 and 22 with EDSS: 2–2.5, all diagnosed with relapsing-remitting MS. All NW-PwMS showed T25FWT scores below the normative cutoff value (<5.2 s) (29). All the instrumented metrics describing natural walking were comparable between NW-PwMS and normative data, and between EDSS subgroups (Table 3). Twenty-nine (49%) NW-PwMS reported that MS had an impact on their walking ability, which was minimal (0 < MSWS-12 ≤ 25) in 18 (30%) and mild (25 < MSWS-12 ≤ 50) in 11 (19%) participants (46). As shown in Table 2, FAB-s and FAB-s-Item6 scores were higher in HS compared to EDSS: 0–1.5 (*p*_BH_ ≤ 0.041) and EDSS: 2–2.5 (*p*_BH_ ≤ 0.016) subgroups. Clinical scores were comparable between EDSS subgroups (Table 2).

**Table 2:** Demographic and clinical characteristics of healthy subjects and normal-walking people with MS.

	**HS (*N* = 40)**	**NW-PwMS (*N* = 59)**	***p*-value**	**EDSS: 0–1.5 (*N* = 37)**	**EDSS: 2–2.5 (*N* = 22)**	***p*-value**
Age (years)	37.5 (24.5; 57)	37 (25; 53)	0.895	35 (25; 55)	40.5 (26; 55)	0.384
Sex (female/male)	28/12	41/18	0.957	25/12	16/6	0.677
Disease duration (years)	-	2 (0; 5)	-	2 (0; 5)	2.5 (0; 5)	0.589
EDSS (0–10)	-	1.5 (0; 2.5)	-	1 (0; 1.5)	2 (2; 2.5)	<0.001
T25FWT (seconds)	-	3.8 (3.2; 5.0)	-	3.8 (3.2; 5.0)	3.8 (3.2; 4.9)	0.857
MSWS-12 (0–100)	-	0 (0; 41.7)	-	0 (0; 41.7)	7.3 (0; 41.7)	0.105
FAB-s (0–24)	24 (23; 24)	23 (19; 24)	<0.001	23 (19; 24)	22 (19; 24)	0.185
FAB-s item 6 (0–4)	4 (4; 4)	4 (2; 4)	<0.001	4 (2; 4)	4 (2; 4)	0.276

*Values are median (5th; 95th percentiles) or number. HS, healthy subjects; NW-PwMS, normal-walking people with MS; EDSS, Expanded Disability Status Scale; T25FWT, Timed 25-foot Walk Test; MSWS-12, 12-item Multiple Sclerosis Walking Scale; FAB-s, Fullerton Advanced Balance scale—short version. p-value: results of the chi-squared test for sex and Mann–Whitney U-test for all the other variables*.

**Table 3:** Instrumented metrics describing fast straight-line walking in normal-walking people with MS (NW-PwMS) and healthy subjects previously tested (NORM).

	**NORM (*N* = 21)**	**NW-PwMS (*N* = 59)**	***p*-value**	**EDSS: 0–1.5 (*N* = 37)**	**EDSS: 2–2.5 (*N* = 22)**	***p*-value**
	***Median (5th−95th percentile)***	***Median (5th−95th percentile)***		***Median (5th−95th percentile)***	***Median (5th−95th percentile)***	
**Spatiotemporal domain**						
Gait speed (m/s)	1.8 (1.4–2.2)	1.7 (1.4–2.0)	0.158	1.7 (1.4–2.0)	1.7 (1.4–1.9)	0.090
Cadence (stride/min)	65.7 (59.0–76.8)	64.9 (59.0–76.9)	0.530	64.9 (59.0–79.6)	64.4 (59.8–70.2)	0.351
Stance duration (%)	53.9 (51.4–57.6)	53.6 (51.2–57.4)	0.460	54.1 (51.2–57.4)	53.3 (51.3–56.5)	0.600
Double support duration (%)	3.3 (0.8–6.6)	3.3 (0.9–6.4)	0.281	3.0 (0.9–6.5)	3.6 (1.1–5.5)	0.567
**Regularity domain**						
AP stride regularity (-)	0.81 (0.60–0.94)	0.80 (0.50–0.94)	0.588	0.80 (0.51–0.96)	0.82 (0.50–0.92)	0.678
ML stride regularity (-)	0.82 (0.66–0.93)	0.81 (0.56–0.94)	0.694	0.82 (0.56–0.96)	0.80 (0.59–0.94)	0.259
**Symmetry domain**						
AP iHR (%)	84.0 (74.5–91.0)	84.7 (69.3–93.2)	0.634	85.0 (69.3–93.7)	84.3 (75.2–91.7)	0.562
ML iHR (%)	90.0 (75.6–91.9)	85.7 (69.0–96.0)	0.170	85.4 (55.9–96.1)	86.7 (71.6–94.1)	0.900
**Local dynamic stability domain**						
AP sLyE (-)	0.75 (0.38–1.40)	0.81 (0.33–1.42)	0.814	0.82 (0.33–1.47)	0.72 (0.35–1.15)	0.562
ML sLyE (-)	0.88 (0.49–1.70)	0.91 (0.33–1.40)	0.706	0.95 (0.26–1.48)	0.81 (0.42–1.17)	0.672
**Trunk sway domain**						
AP normalized acceleration (-)	0.48 (0.41–0.57)	0.47 (0.34–0.60)	0.548	0.46 (0.33–0.60)	0.49 (0.41–0.55)	0.170
ML normalized acceleration (-)	0.52 (0.38–0.62)	0.54 (0.38–0.82)	0.235	0.56 (0.33–0.86)	0.51 (0.43–0.70)	0.100

*AP, anteroposterior; ML, mediolateral; iHR, improved harmonic ratio; sLyE, short-term Lyapunov exponent. p-value: results of the Mann–Whitney U-tests with Bonferroni–Holm correction for multiple comparisons*.

### Instrumented WHHT: HS vs. NW-PwMS

As reported in Table 4, spatiotemporal parameters and trunk sway during WHHT were comparable between NW-PwMS and HS and showed poor discriminant ability (0.52 ≤ AUC ≤ 0.58). ML stride regularity and AP gait symmetry (AP iHR) were lower in NW-PwMS compared to HS. ML dynamic stability was reduced (higher ML sLyE) in NW-PwMS compared to HS. These three metrics showed moderate discriminant ability (AUC ≥ 0.71) and were therefore considered for the subsequent analyses.

**Table 4:** Instrumented metrics describing walking with horizontal head turns in healthy subjects and normal-walking people with MS.

	**HS (*N* = 40)**	**NW-PwMS (*N* = 59)**	***p*-value**	**AUC**
	***Median (5th; 95th percentile)***	***Median (5th; 95th percentile)***		***Mean (95% CI)***
**Spatiotemporal domain**				
Gait Speed (m/s)	0.93 (0.66; 1.22)	0.89 (0.54; 1.20)	0.199	0.57 (0.46; 0.69)
Cadence (stride/min)	51.2 (42.1; 56.8)	50.5 (38.6; 54.6)	0.164	0.58 (0.46; 0.69)
Stance dur. (%)	57.3 (53.7; 61.9)	57.6 (53.9; 62.1)	0.445	0.53 (0.42; 0.65)
Double-support dur. (%)	7.4 (4.0; 11.7)	7.6 (4.0; 11.3)	0.295	0.55 (0.44; 0.67)
**Regularity domain**				
AP stride regularity (-)	0.66 (0.33; 0.88)	0.60 (0.17; 0.84)	0.109	0.64 (0.53; 0.75)
ML stride regularity (-)	0.68 (0.52; 0.84)	0.54 (0.10; 0.81)	**0.001**	0.71 (0.61; 0.81)
**Symmetry domain**				
AP iHR (%)	78.0 (67.3; 89.4)	73.3 (50.8; 81.8)	**<0.001**	0.73 (0.63; 0.83)
ML iHR (%)	72.4 (51.6; 87.1)	69.4 (51.8; 87.3)	0.239	0.57 (0.46; 0.68)
**Dynamic stability domain**				
AP sLyE (-)	0.71 (0.33; 1.14)	0.76 (0.36; 1.32)	0.347	0.55 (0.44; 0.67)
ML sLyE (-)	0.53 (0.26; 0.78)	0.66 (0.39; 1.06)	**<0.001**	0.73 (0.64; 0.83)
**Trunk sway domain**				
AP norm. trunk acc. (-)	0.41 (0.27; 0.57)	0.39 (0.26; 0.61)	0.719	0.52 (0.40; 0.64)
ML norm. trunk acc. (-)	0.40 (0.29; 0.55)	0.43 (0.31; 0.69)	0.363	0.54 (0.42; 0.66)

*HS, healthy subjects; NW-PwMS, normal-walking people with MS; AUC, area under the receiver operating characteristic curve; AP, anteroposterior; ML, mediolateral; iHR, improved harmonic ratio; sLyE, short-term Lyapunov exponent. p-value: results of the Mann–Whitney U-test with Bonferroni–Holm correction for multiple comparisons. Statistically significant (p < 0.05) results are reported in bold*.

**Figure 1:**
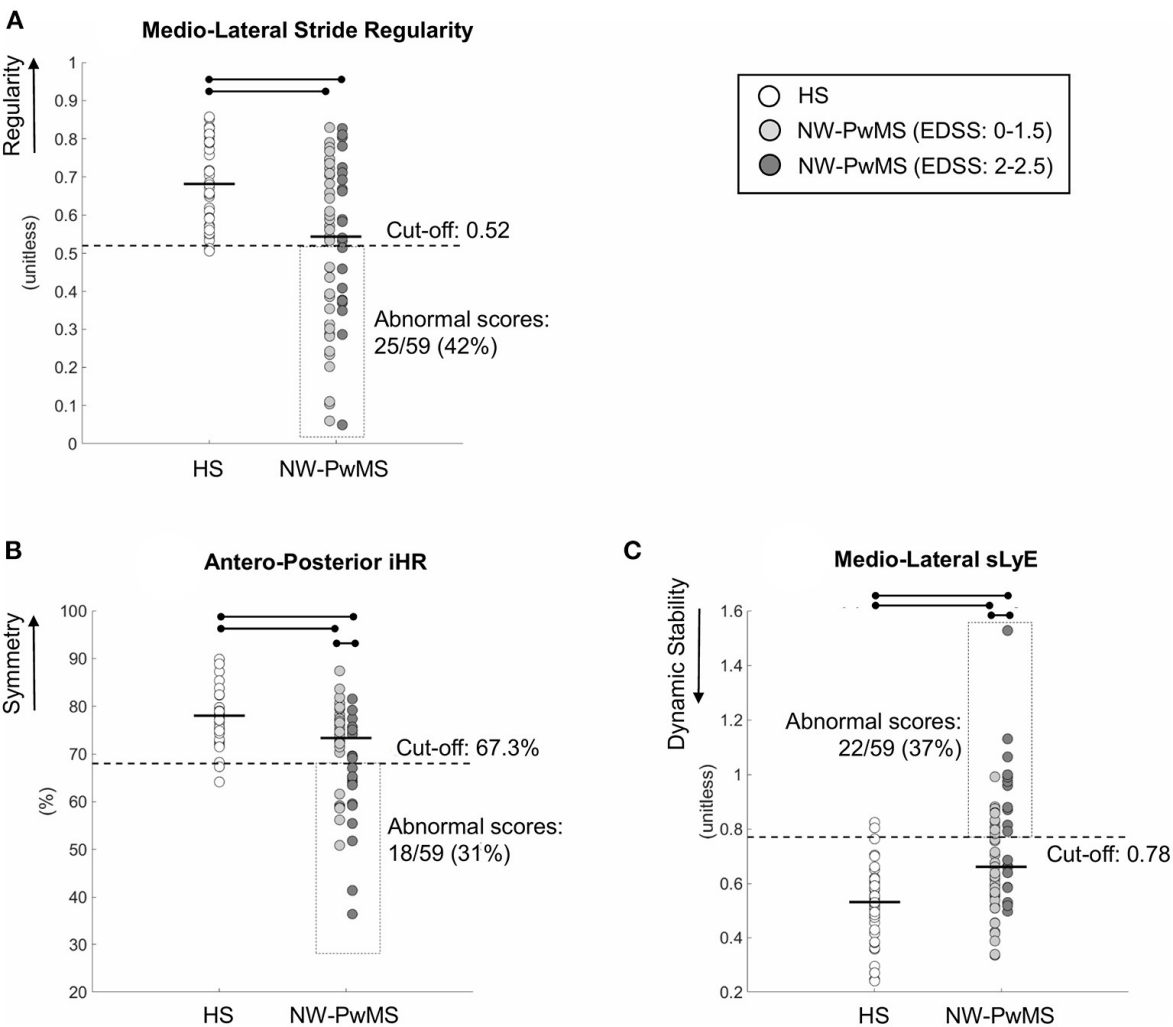
Instrumented parameters describing walking with horizontal head turns (WHHT) in healthy subjects (HS) and early-stage people with MS showing normal walking (NW-PwMS). **(A)** Mediolateral Stride Regularity. **(B)** Anteroposterior iHR (improved harmonic ratio). **(C)** Mediolateral sLyE (short-term Lyapunov exponent). Each circle represents a single participant. Horizontal bold lines represent median values for each group. Horizontal lines and dots represent a statistically significant difference between groups (*p* < 0.05, Kruskal–Wallis and Bonferroni–Holm test). Cut-off scores, corresponding to the 5th percentile **(A,B)** and the 95th percentile **(C)** of HS, are reported together with the number (%) of NW-PwMS showing abnormal values.

The number of NW-PwMS showing abnormal values was 25 (42%) for ML stride regularity, 18 (31%) for AP iHR, and 22 (37%) for ML sLyE (Figure 1).

### Instrumented WHHT: HS vs. EDSS: 0–1.5 vs. EDSS: 2–2.5

Significant differences between HS and EDSS subgroups were found (*p*_KW_ < 0.001). ML regularity, AP symmetry, and ML dynamic stability were higher in HS compared to EDSS: 0–1.5 (*p*_BH_ ≤ 0.027) and EDSS: 2–2.5 (*p*_BH_ ≤ 0.034) subgroups (Figure 1). ML regularity was comparable between EDSS subgroups (*p*_BH_ = 0.490). EDSS: 0–1.5 subgroup showed higher AP symmetry (*p*_BH_ = 0.019) and ML dynamic stability (i.e., lower ML sLyE) (*p*_BH_ = 0.026) than EDSS: 2–2.5 subgroup (Figure 1). The discriminant ability was moderate [AUC mean (95% CI) AP symmetry: 0.70 (0.56–0.84); ML sLyE: 0.69 (0.55–0.84)].

The number of participants showing abnormal values of ML regularity was comparable between EDSS subgroups [EDSS: 0–1.5: 16/37 (43%); EDSS: 2–2.5: 9/22 (41%); *p*_χ2_ = 0.861]. A larger number of EDSS: 2–2.5 vs. EDSS: 0–1.5 NW-PwMS showed abnormal scores of ML dynamic stability [12/22 (55%) vs. 10/37 (27%); *p*_χ2_ = 0.035] and AP symmetry [12/22 (55%) vs. 6/37 (16%); *p*_χ2_ = 0.002].

### Instrumented WHHT vs. Clinical Scales

**Figure 2:**
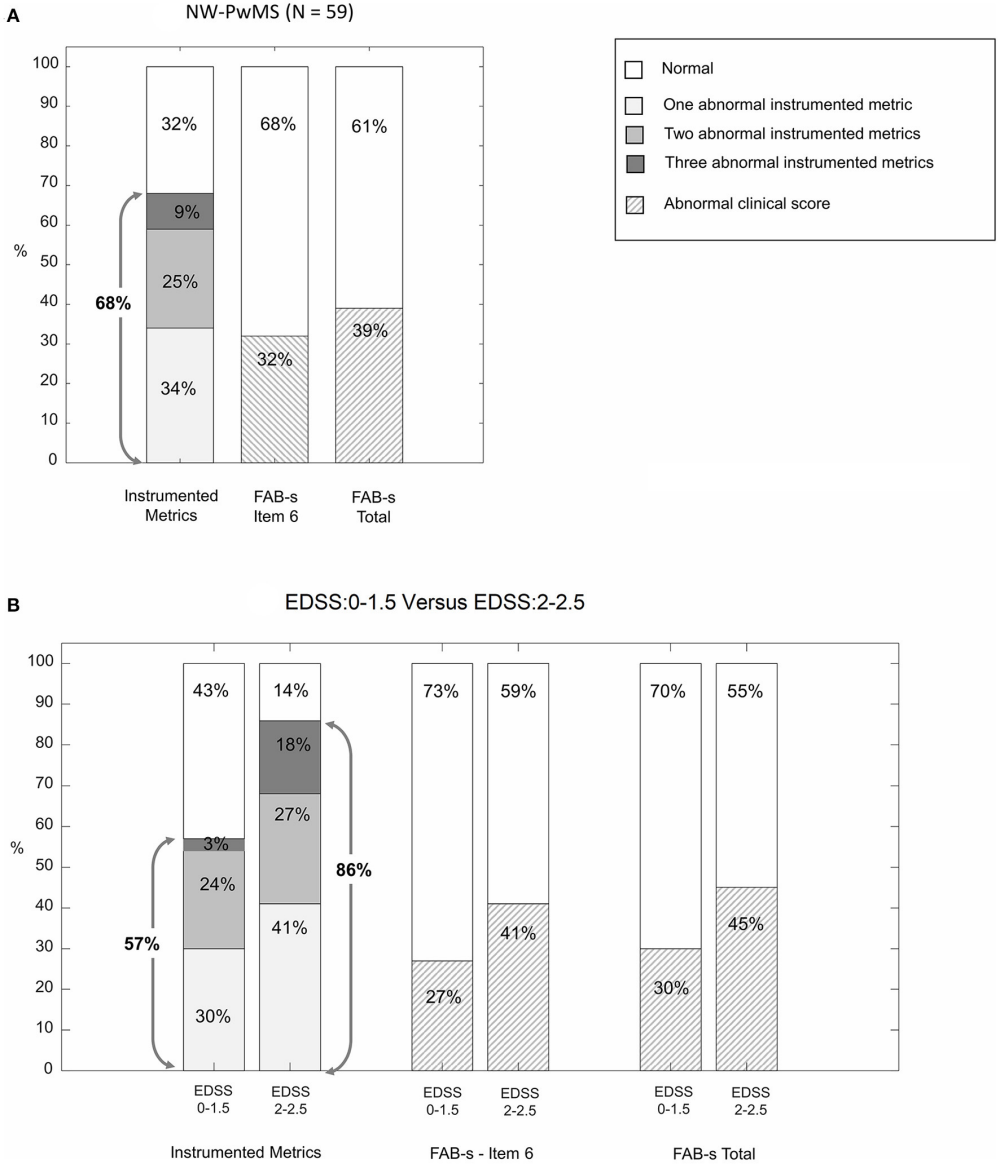
Percentage of normal-walking people with MS (NW-PwMS) showing abnormal values of instrumented metrics descriptive of walking with horizontal head turns (WHHT) and abnormal clinical scores on FAB-s (Fullerton Advanced Balance scale-short version). **(A)** Whole sample of NW-PwMS. **(B)** NW-PwMS sub-samples showing, respectively, EDSS: 0–1.5 and EDSS: 2–2.5. Gray arrows indicate the percentage of NW-PwMS showing at least one abnormal instrumented metric.

Figure 2A reports the percentages of NW-PwMS showing abnormal instrumented metrics (ML regularity, AP symmetry, and ML dynamic stability) and abnormal FAB-s scores (<23) and FAB-s-Item6 subscores (<4). Forty NW-PwMS (68%) showed at least one abnormal instrumented metric. This percentage was larger than those representing individuals with abnormal FAB-s-Item6 subscore [19/59 (32%), *p*_χ2_ < 0.001] and FAB-s score [23/59 (39%), *p*_χ2_ = 0.002].

The number of individuals presenting at least one abnormal instrumented metric was larger (*p*_χ2_ = 0.019) in EDSS: 2–2.5 [19/22 (86%)] vs. EDSS: 0–1.5 subgroup [21/37 (57%)] (Figure 2B). The same trend was found in clinical scales (Figure 2B), although not statistically significant (*p*_χ2_ ≥ 0.226).

### Correlation Analysis and Concurrent Validity

ML stride regularity, AP iHR, and ML sLyE showed low non-significant correlations between each other (−0.23 ≤ *r*_*s*_ ≤ 0.17, *p*_BH_ ≥ 0.222).

As shown in Table 5, statistically significant correlations were found between the three instrumented metrics and FAB-s and FAB-s-Item 6 scores. ML sLyE moderately correlated also with MSWS-12.

**Table 5:** Spearman's correlation coefficient (*r*_*s*_) between instrumented metrics and clinical scores.

	**FAB-s Item 6 subscore**	**FAB-s score**	**MSWS-12 score**
ML stride regularity (-)	0.37^***^	0.46^***^	−0.13
AP iHR (%)	0.39^***^	0.49^***^	−0.25
ML sLyE (-)	−0.44^***^	−0.48^***^	0.34^*^

*AP, anteroposterior; ML, mediolateral; iHR, improved harmonic ratio; sLyE, short-term Lyapunov exponent; FAB-s, Fullerton Advanced Balance scale—short version; MSWS-12, 12-item Multiple Sclerosis Walking Scale*.

* *p < 0.05;*

*** *p < 0.001 (Bonferroni–Holm correction)*.

## Discussion

A wearable-sensor-based assessment of WHHT was applied to HS and early-stage NW-PwMS to evaluate the presence of subclinical impairments not detected by clinical and instrumented measures of natural walking. This would help clinicians to discriminate between individuals with normal and abnormal dynamic balance, to identify, from the very early stages of the disease, those persons who may benefit from preventive rehabilitation exercise, and to track subtle impairments over the disease course. Three IMU-derived metrics, descriptive of regularity, symmetry, and stability of WHHT, were significantly impaired in NW-PwMS compared to HS and were able to discriminate between EDSS-based subgroups. The discriminant ability of the instrumented metrics was higher compared to FAB-s-Item6 and FAB-s clinical scores, and the significant correlations with the clinical scales demonstrated their concurrent validity.

Walking impairment is a hallmark of MS developing early in the disease course. Previous studies on walking in early-stage PwMS found altered spatiotemporal parameters (23, 31, 47), abnormal trunk sway (22), and increased variability (36, 48), instability (37, 38), and asymmetry (39, 49) compared to HS. No such abnormalities were found in the present cohort of NW-PwMS, at least during short-distance walking tests. The sample can thus be considered composed of PwMS showing normal natural locomotion, as highlighted also by the high gait speed derived from the T25FWT (1.9 ± 0.3 m/s) that is comparable with the mean velocity (1.8 ± 0.3 m/s) obtained from 31 studies, analyzed in a recent review (50), on the T25FWT in healthy subjects. Despite these results, the MSWS-12 scores indicated that 49% of participants perceived that MS influenced their walking capacity, at least minimally. Moreover, the FAB-s score was significantly reduced compared to HS, confirming that dynamic balance impairment is an early disease-related sign (46).

Regarding WHHT, both the FAB-s-Item6 sub-score and the instrumented parameters revealed significant anomalies in NW-PwMS vs. HS. In particular, ML regularity, AP symmetry, and ML dynamic stability were reduced in 31–42% of NW-PwMS and showed a moderate discriminant ability. Importantly, the three features were not correlated with each other, suggesting the presence of subclinical impairments affecting independent locomotor domains. Interestingly, the present results revealed subtle impairments also in the EDSS: 0–1.5 subgroup. Since both the present results on straight-line walking and previously published results on instrumented TUG (51) did not reveal abnormalities in EDSS: 0–1.5 patients, it can be suggested that the instrumented assessment of WHHT may be a more sensitive tool (than those mentioned above) to identify, already from the very early phases of the disease, incipient balance and locomotor anomalies that become clinically evident only in the most advanced stages of MS (EDSS≥4) (52–55).

The abnormalities found during WHHT could be primarily ascribed to the significant impairment of dynamic balance. The FAB-s score was abnormal in 39% of NW-PwMS and significantly correlated with the three instrumented metrics, indicating that poorer balance was associated with lower regularity, symmetry, and stability during WHHT. Although the Kurtzke Functional Systems scores (in particular Pyramidal, Cerebellar, Brainstem, and Sensory scores) (28) have not been addressed in this study, it can be speculated that sensory loss, a typical early sign of MS (25), may have been a significant factor affecting balance. Particularly, somatosensory and proprioceptive impairments may have increased the reliance on the vestibular system that could show alterations also in early-stage PwMS (56), especially when challenged during WHHT. Also, the possible impairments of the pyramidal system, representing the first clinical sign of MS in 22% of patients (57), may have played a role in reducing balance, as previously demonstrated by Martin et al. on early-stage PwMS (58), and in increasing step asymmetry, as found by Kalron and Givon on more severe patients (59). Another aspect that may be considered is that WHHT is, actually, a dual-task requiring attention to turn the head at the metronome beat while walking. Previous studies on PwMS have demonstrated that different dual-task paradigms adversely affect balance and walking also in early-stage subjects (60). This, in turn, may further explain the presence of abnormal WHHT patterns, even in participants with normal (single-task) walking.

Interestingly two of the three selected metrics (regularity and stability) were abnormal in ML direction. Previous studies on PwMS have demonstrated that several ML parameters descriptive of balance (61, 62) are more altered in fallers vs. non-fallers. Considering that falls/near falls have been reported in 30% of early-stage PwMS (46), future studies should assess if the WHHT metrics could be predictive of fall risk also in this population.

While the clinical scores were comparable between EDSS subgroups, the instrumented WHHT revealed that the EDSS: 2–2.5 subgroup was characterized by lower AP symmetry and reduced ML dynamic stability compared to the EDSS: 0–1.5 subgroup. This indicated that the instrumented assessment of WHHT could be a sensitive tool to detect differences also between subgroups of PwMS in the lower range of EDSS. These findings suggest that AP symmetry and ML dynamic stability describing WHHT could be responsive indexes to monitor the disease progression. Further longitudinal studies including subjects with a larger spectrum of disability should be performed to corroborate this hypothesis.

Compared to the FAB-s clinical scores, the instrumented WHHT demonstrated a higher ability to discriminate between HS and NW-PwMS: the percentage of participants showing at least one abnormal instrumented metric (68%) was statistically larger than that detected by FAB-s-Item6 subscore (32%) and FAB-s score (39%). This result was found also considering separately the two EDSS subgroups, further supporting the larger sensitivity of the instrumented WHHT. Finally, the correlation analysis between the FAB-s scores and the instrumented metrics revealed a moderate concurrent validity of the proposed indexes to measure dynamic balance impairments. Interestingly, ML dynamic stability, as measured by sLyE, was significantly correlated with the MSWS-12. This finding complements previous results showing that balance dysfunctions and instability are major contributors to the perceived MS-related walking disturbances also in the early stage of MS (39, 46). This result, together with previous findings of the responsiveness of sLyE to rehabilitation (24) and its association to fall risk in PwMS (55), suggests that this parameter, in particular, could be a promising sensitive biomarker to monitor the disease course from the beginning of MS and that exercises aimed at improving dynamic balance and stability should be proposed also to early-stage, high functioning PwMS. Future studies are necessary to confirm this hypothesis.

### Study Limitations

First, the proposed instrumented metrics were computed on five strides that are those required by the FAB-s instructions but are less than those suggested to increase the robustness of the parameters (10–20 strides) (41, 63). However, the use of a test already validated is undoubtedly an advantage because of its clinical application. Future studies considering more consecutive strides or more repetitions of short walking bouts (64) should be performed to assess the test-retest reliability of the instrumented WHHT. Second, the Functional Systems scores have not been addressed since one of the aims of this study was to compare subgroups of PwMS with different EDSS global scores, independently from the functional systems involved. Third, although hearing loss is considered a rare symptom of MS, it is not uncommon (65). Even if none of the participants reported auditory problems, a dedicated exam was not performed. Hence, considering that the subjects had to turn their head at a metronome beat, we cannot exclude a possible influence of eventual hearing loss on the results. Further studies should address this aspect and the possible effect of the different functional systems. Finally, the tested sample consisted of early-stage high-functioning PwMS, thereby reducing the generalizability of present results.

## Conclusion

The present results confirmed our hypotheses: the IMU-based assessment of WHHT provides valid objective metrics able to discriminate, with a higher sensitivity than clinical scores, between HS and NW-PwMS and between EDSS subgroups. The method is a promising tool to complement clinical assessments and detect subtle impairments in early-stage non-disabled PwMS who still show normal natural walking. This approach would help in tracking these impairments over time and identifying those individuals who may benefit from preventive motor exercise since the very early stages of MS, when rehabilitation may still have neuroprotective and disease-modifying effects, as recently suggested (26). Future studies, including more severe PwMS, are warranted to assess the reliability and the clinical responsiveness of the proposed metrics.

## Data Availability Statement

The raw data supporting the conclusions of this article will be made available by the authors, without undue reservation.

## Ethics Statement

The studies involving human participants were reviewed and approved by the local Ethical Committee of each center. These were: Comitato Etico della Sezione “IRCCS Fondazione Don Carlo Gnocchi” del Comitato Etico IRCCS Regione Lombardia (IRCCS Fondazione Don Carlo Gnocchi, Milan, Italy), Comitato Etico Interaziendale dell'A.O “SS. Antonio e Biagio e Cesare Arrigo” (Centro di Recupero e Rieducazione Funzionale Mons. Luigi Novarese, Moncrivello, Italy), and Comitato Etico Regionale della Liguria (Italian Multiple Sclerosis Foundation, Genoa, Italy). The patients/participants provided their written informed consent to participate in this study.

## Author Contributions

Conception and design of the study, software implementation, data processing, data analysis, data interpretation, and drafting the manuscript by IC. Instrumented data collection, clinical assessment, and data organization by EG, DA, and RD. Data collection and organization by AT. Recruitment of patients and clinical assessment by GB, PC, CS, and MR. Conceptualization and design of the study, data analysis and interpretation, and coordination by DC. Conceptualization and design of the study and coordination by MF, CS, and GB. All authors contributed to data interpretation, critically reviewed the manuscript, and approved the final version of the manuscript.

## Funding

This study was supported by the Italian Multiple Sclerosis Foundation—FISM (FISM grant 2016, N16/17/F14) and by the Italian Ministry of Health (Fondi di Ricerca Corrente).

## Conflict of Interest

The authors declare that the research was conducted in the absence of any commercial or financial relationships that could be construed as a potential conflict of interest.

## Publisher's Note

All claims expressed in this article are solely those of the authors and do not necessarily represent those of their affiliated organizations, or those of the publisher, the editors and the reviewers. Any product that may be evaluated in this article, or claim that may be made by its manufacturer, is not guaranteed or endorsed by the publisher.
